# Supplementary material for: Self-Powered, Non-Toxic, Recyclable Thermogalvanic Hydrogel Sensor for Temperature Monitoring of Edibles
Source: Micromachines (Basel). 2023 Jun 28;14(7):1327. doi: 10.3390/mi14071327 (PMC10385118; doi:10.3390/mi14071327)
Supplement: Supplementary file 1 [file micromachines-14-01327-s001.zip › micromachines-2466462-supplementary.pdf]

Supporting Information

Self-Powered, Non-Toxic, Recyclable Thermogalvanic Hydrogel Sensor for Temperature Monitoring of Edibles

Kun Yang [1,*], Chenhui Bai [1], Boyuan Liu [1], Zhoutong Liu [1] and Xiaojing Cui [2,3,*]

1 College of electronic information and optical engineering, Taiyuan University of Technology, Taiyuan, 030024, China

2 Shanxi Transportation Technology Research & Development Co., Ltd., Taiyuan, 030032, China

3 School of Physics and Information Engineering, Shanxi Normal University, Taiyuan,030031, China

***** Correspondence: yangkun01@tyut.edu.cn (K.Y.); 20210084@sxnu.edu.cn (X.C.)

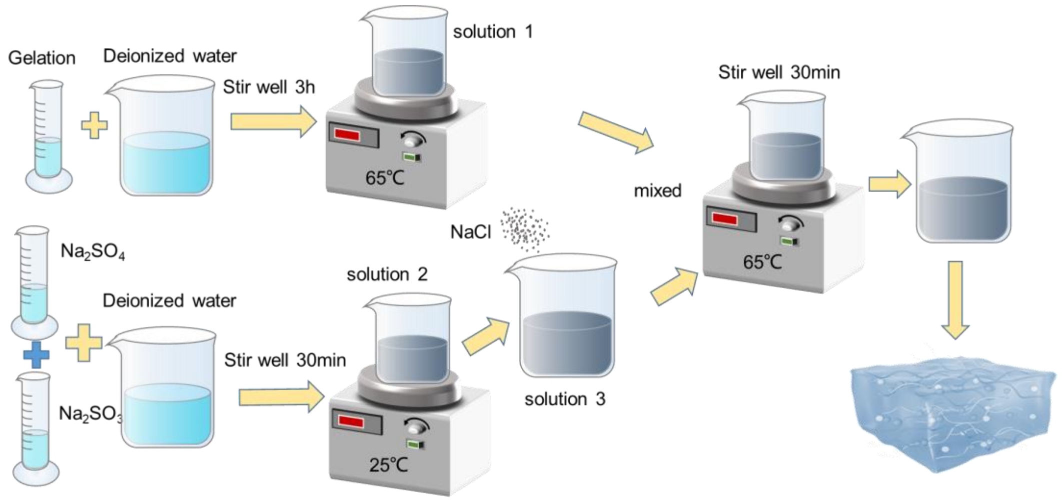

Figure S1. Preparation process of Gel- $SO_{3/4}^{2}$ gel

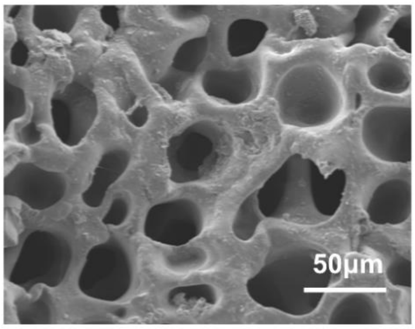

Figure S2. SEM image of a Gelatin gel after freeze-drying.

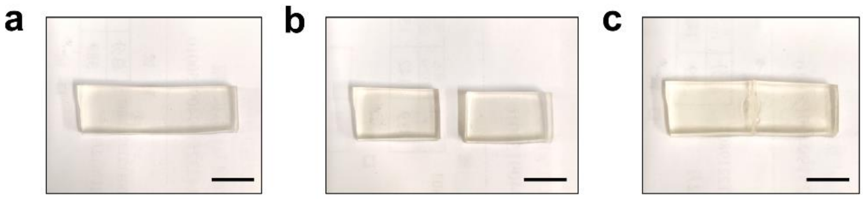

Figure S3. Self-healing photo of Gel-$SO_{3/4}^{2-}$ gel.

(a) Initial state; (b) Cut off status; (c) Healing status.

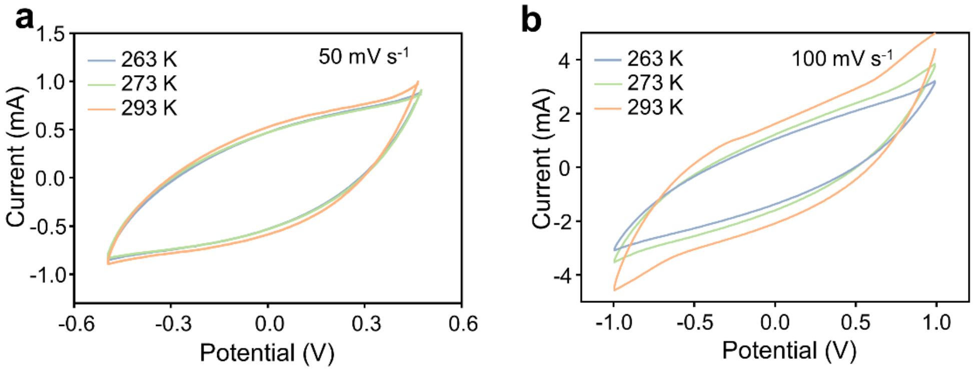

Figure S4. Cyclic voltammetry (CV) curves scanned at different temperatures.

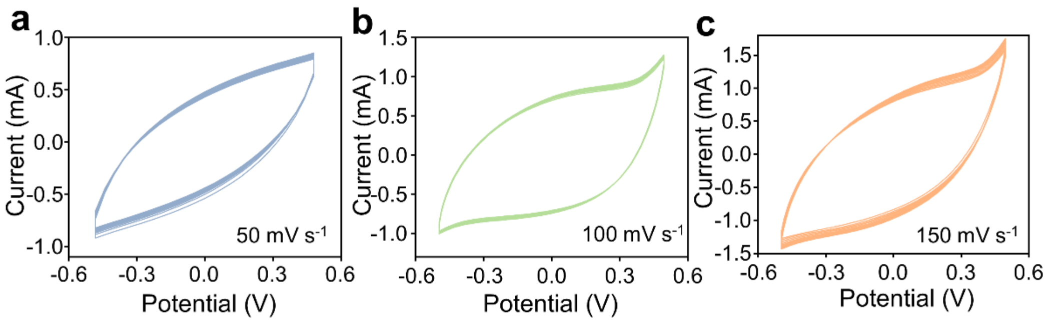

Figure S5 Cyclic voltammetry (CV) curves scanned at 50 mV s^{-1} (a), 100 mV s^{-1} (b), 150 mV s^{-1} (c), respectively.

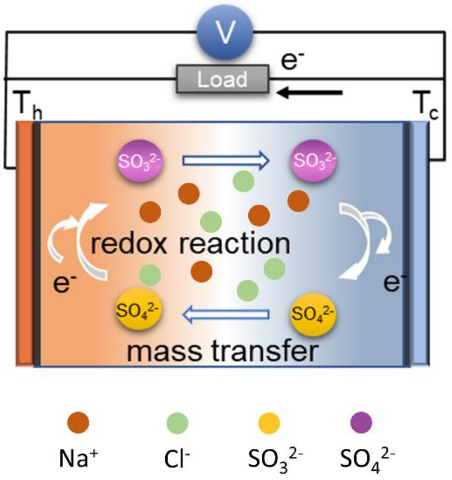

Figure S6. The overall working mechanism and equivalent circuit.

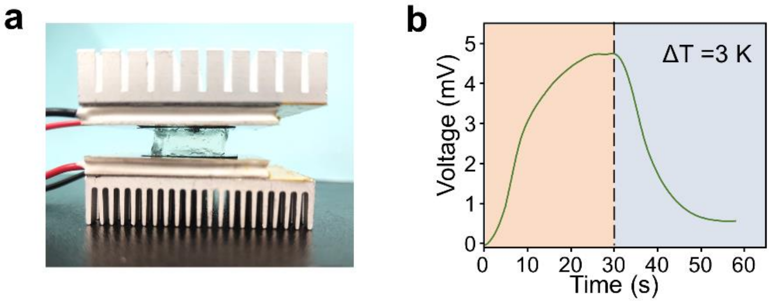

Figure S7. (a) Physical photo of thermoelectric measurement platform.

(b) Time-voltage curve for a temperature difference of 3K between the two ends of the gel.

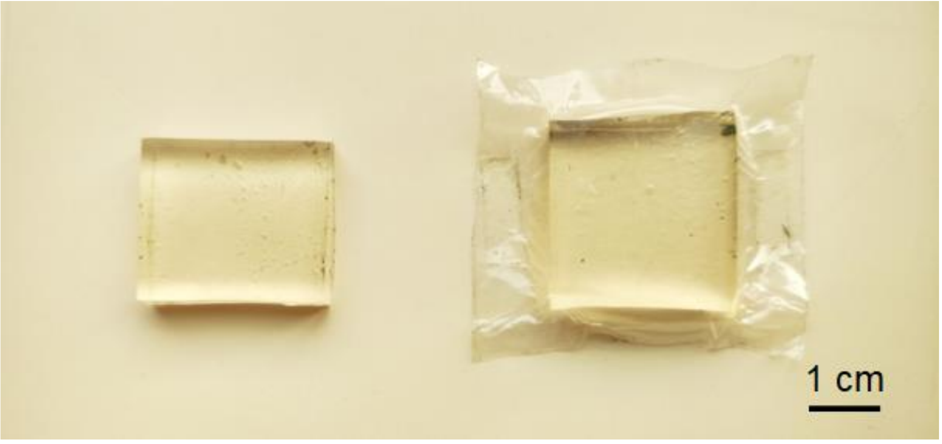

Figure S8. Gel-$SO_{3/4}^{2-}$ gel encapsulated and unencapsulated optical photos

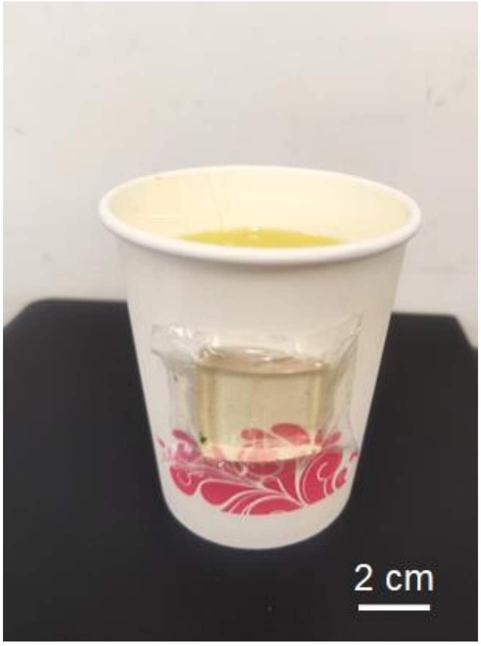

Figure S9. Gel-$SO_{3/4}^{2-}$ gel for measuring water temperature.

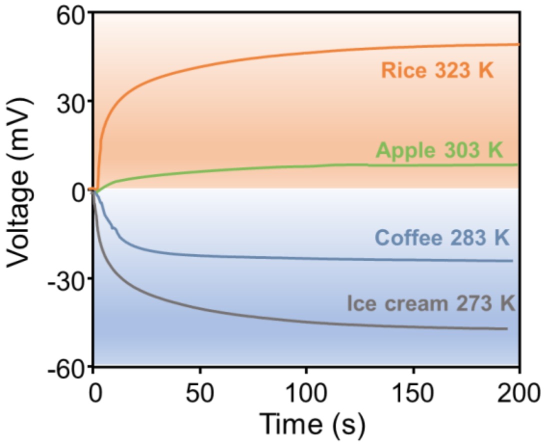

Figure S10. Gel-$SO_{3/4}^{2-}$ gel for temperature detection of different foods.
